# Supplementary material for: Targeted dimensionality reduction enables reliable estimation of neural population coding accuracy from trial-limited data
Source: PLoS One. 2022 Jul 21;17(7):e0271136. doi: 10.1371/journal.pone.0271136 (PMC9302847; doi:10.1371/journal.pone.0271136)
Supplement: S1 Appendix — (PDF) [file pone.0271136.s001.pdf]

Variance of parameter estimates

In this work, we approximate the spike counts of a neural population as being drawn from a multivariate Gaussian with mean μ and covariance Σ. The accuracy of our estimates of these respective parameters depends on how large the sample size is. That is, if we draw just two samples from the distribution $\mathcal{N}(\mu, \Sigma)$, our estimates of μ and Σ will be highly variable across repeated iterations of this sampling. This means that when sample size is small we can't be certain of the measured parameter values. Here, we provide a brief derivation showing how the uncertainty in each of these parameter values depends on sample size, k.

Mean (μ):

We will investigate the mean of just a single neuron, μ, for simplicity. Here, and in the following cases, we assume the data has been centered such that the mean response across all trials for each neuron is zero. Consider repeated samples of a random variable, x_i, drawn from $\mathcal{N}(0, \sigma^2)$. Let us define the variable Y to be the mean of a random sequence of i.i.d. numbers, $x_i ... x_n$ with $E[x_i] = \mu$ and $Var(x_i) = \sigma^2$.

$$Y = \frac{1}{k} \sum_{i=1}^{k} x_i$$

Next, we can ask how *variable* our estimates of Y are with increasing sample size.

$$Var(Y) = Var\Big(\frac{1}{k} \sum_{i=1}^{k} x_i\Big)$$

$$Var(Y) = \frac{1}{k^2} \sum_{i=1}^{k} Var(x_i)$$

$$Var(Y) = \frac{1}{k^2} \sum_{i=1}^{k} \sigma^2$$

$$Var(Y) = \frac{\sigma^2}{k}$$

Thus, estimates of the mean spike count for a single neuron, μ, decay with increasing sample size as:

$$\mathcal{O}\big(\frac{1}{k}\big) \tag{7}$$

Single neuron variance (Σ_{diag}):

For the variance of single neurons, *i.e.* the diagonal elements of Σ, we can similarly derive their uncertainty as a function of k by defining Y as:

$$Y = \frac{1}{k-1}\sum_{i=1}^{k} x_i^2$$

$$Var(Y) = Var\Big(\frac{1}{k-1}\sum_{i=1}^{k} x_i^2\Big)$$

$$Var(Y) = \frac{1}{(k-1)^2}\sum_{i=1}^{k} Var(x_i^2)$$

$$Var(Y) = \frac{1}{(k-1)^2}\sum_{i=1}^{k} 2\sigma^4$$

$$Var(Y) = \frac{2\sigma^4}{k-1}$$

Thus, the uncertainty in single neuron variance depends the neuron's true variance σ^2, and decays as a function of sample size k.

$$\mathcal{O}(\frac{1}{k-1}) \tag{8}$$

Covariance (Σ):
And finally, for uncertainty of the covariance between two correlated neurons x and y, *i.e.* the off-diagonal elements of Σ, we define Y as:

$$Y = \frac{1}{k-1}\sum_{i=1}^{k} x_i y_i$$

As above, can write:

$$Var(Y) = Var\Big(\frac{1}{k-1}\sum_{i=1}^{k} x_i y_i\Big)$$

$$Var(Y) = \frac{1}{(k-1)^2}\sum_{i=1}^{k} Var(x_i y_i)$$

Then, using the three following identities:

$$Var(xy) = E[x^2y^2] - E[xy]^2$$
$$E[x^2y^2] = cov(x^2, y^2) + E[x^2]E[y^2]$$
$$E[XY]^2 = \big(cov(x,y) + E[x]E[y]\big)^2$$

We can write the following expression for the $Var(Y)$, taking $E[x] = E[y] = 0$:

$$Var(Y) = \Big(\frac{2(\Sigma_{x,y}^2)^2 + \sigma_x^2\sigma_y^2 - \Sigma_{x,y}}{k-1}\Big)$$

where $\Sigma_{x,y}$ is the true covariance between neurons x and y, and σ_x^2 and σ_y^2 represent each neuron's respective independent variance. Thus, as for single neuron variance, the uncertainty in covariance decays with sample size, k (Eqn. 9). Note, though, that typical covariance values are much smaller than single neuron variance, making this a much more difficult parameter to estimate given a particular sample size.

$$\mathcal{O}\Big(\frac{1}{k-1}\Big) \tag{9}$$
